# Macromolecular High‐Affinity Binding Probed by Advanced Fluorescence Techniques

**DOI:** 10.1002/cbic.202500283

**Published:** 2025-07-24

**Authors:** Alida Meyer, Benno Schedler, Jörg Fitter

**Affiliations:** ^1^ RWTH Aachen University I. Physikalisches Institut (IA) AG Biophysik 52074 Aachen Germany; ^2^ ER‐C‐3 Structural Biology Forschungszentrum Jülich 52425 Jülich Germany

**Keywords:** biosensors, fluorescence spectroscopy, FRET, protein‐protein interactions, single‐molecule studies

## Abstract

Due to the extreme sensitivity and the intrinsic selectivity of fluorescence techniques, high‐affinity binding can be measured even at extremely low molecule concentrations in the picomolar range. In particular, modern advanced techniques with fluorescence microscopes have provided considerable methodological advancements in recent years. Here, a brief description of the basic physical principles of fluorescence detection and its experimental measurement setups are provided. For interacting biomolecules in solution, confocal fluorescence microscopy enables some very effective approaches to characterize binding in complex sample environments and with small sample consumption. In addition to standard techniques with bulk samples in classical spectrometers, applications with single‐molecule Förster resonance energy transfer, two‐color coincidence detection, and fluorescence correlation spectroscopy are presented. The strength of the more advanced techniques lies in their broad applicability, ranging from fluorescence‐based genetically encoded biosensors for use in living cells to the high controllability in the measurement of binding curves even at very low molecule concentrations. The advantages and limitations of the individual techniques are compared and recent state‐of‐the‐art applications are discussed.

## Graphical abstract

For interacting biomolecules in solution, confocal fluorescence microscopy enables some very effective approaches to characterize intermolecular binding in complex sample environments and with small sample consumption. These approaches range from fluorescence‐based genetically encoded biosensors for use in living cells to the high controllability in measuring binding curves even at very low picomolar concentrations.

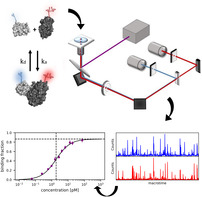

## 1 Introduction

For a detailed understanding of the inter‐molecular binding between bio‐macromolecules, but also between macromolecules and ligands, it is essential to study the molecular interactions in terms of binding stoichiometry, specificity, affinity, and cooperativity.^[^ ^1^ ^]^ In this context, in addition to the values of the equilibrium binding constant (K_D_) and association and dissociation rates (k_a_, k_d_), thermodynamic parameters that describe the character of the binding (Δ*G*, Δ*H*, Δ*S*, Δ*C*_p_) are also of interest.^[^ ^2^ ^]^ A variety of experimental techniques are available to investigate the binding between two biomolecules. In many of these methods the target and the titrant molecules are mixed in different molar ratios to establish a binding equilibrium, in which the bound complexes and the unbound components are quantified^[^ ^3^ ^]^ (see Box 1). For such an approach, numerous methods have been developed, including calorimetric methods, optical methods, and gel filtration methods.^[^ ^1^, ^4^ ^]^ Here, the most commonly used methods are given by isothermal titration calorimetry (ITC),^[^ ^5^ ^]^ surface plasmon resonance (SPR),^[^ ^6^ ^]^ and biolayer interferometry (BLI),^[^ ^7^ ^]^ electrophoretic mobility shift assay (EMSA)^[^ ^8^ ^]^ as well as several fluorescence‐based methods.^[^ ^9^ ^]^ The possible techniques typically differ in their (i) range of application in terms of affinity (e.g., low affinity: μM to a hundred nM versus high affinity: few 10 nM‐pM), by (ii) their properties of the binding partners in the sample (e.g., “surface tethered” vs “freely diffusing in solution”), or (iii) by the necessity of special sample preparation (e.g., fluorescent dye labeling required or not).

**Box 1: Bi‐Molecular Binding**

1 For a bi‐molecular binding one assumes that one binding partner, the ligand *L,* can bind to another one, for example a protein *P,* to form a protein‐ligand complex *P · L.* The reversible binding process

(1) $$P\;+\;L\;\overset{k_{a}}{\underset{k_{d}}{⇄}}\;P\cdot L$$

is characterized by the association rate *k*_a_ and the dissociation rate *k*_d_. For equilibrium the dissociation constant *K*_*D*_ and the association constant *K*_*A*_ are defined by

(2) $$K_{D}\;=\;\frac{k_{d}}{k_{a}}\;=\;\frac{1}{K_{A}}\;=\;\frac{{\left[P\right]}_{free}\;\cdot {\left[L\right]}_{free}}{\left[P\cdot L\right]}$$

where [*P*]_*free*_, [*L*]_*free*_, and [*P* · *L*] denote the concentration of unbound protein, unbound ligand, and bound protein‐ligand complexes, respectively. The physical parameter that is usually employed to characterize the equilibrium is given by the binding fraction using the hyperbolic model

(3) $$f\;=\;\frac{\left[P\cdot L\right]}{{\left[P\right]}_{total}}\;=\;\frac{\left[P\cdot L\right]}{{\left[P\right]}_{free}\;+\;[P\cdot L]}\;=\;\frac{{\left[L\right]}_{free}}{{\left[L\right]}_{free}\;+\;K_{D}}$$

Approaches, employing both binding partners labeled with differently colored dyes, allow to quantify the concentration of all ingredients explicitly (i.e., [*P*]_*free*_, [*P*]_*total*_, [*L*]_*free*_ ,[*L*]_*total*_, and [*P* *· L*]). If one assumes *L* is labeled with a red dye (where [*L*]_*total*_ is for example calculated with *N*_*R*_) we obtain [*L*]_*free*_ values by using one of the two possible formulas

(4) $$\begin{array}{c} {\left[L\right]}_{free}\;=\;{\left[L\right]}_{total}\;-\;f_{BR}\cdot {\left[P\right]}_{total}\\ {\left[L\right]}_{free}\;=\;{\left[L\right]}_{total}\;\cdot \;\;\left(1-f_{BR}\right) \end{array}$$

where the involved coincidence fractions *f*_*BR*_ and *f*_*RB*_ are defined and explained in Section 3.4. To determine *K*_*D*_, typically the concentration of [*P*]_*total*_ is fixed to a certain value and *[L]* is varied with 0.1 *K*_*D*_ < *[L] <* 10 *K*_*D*_. However, when measuring the binding curve, one has to choose the concentration of [*P*]_*total*_ carefully. Only with [*P*]_*total*_ << *K*_*D*_ (i.e., “binding” regime) one obtains a correct *K*_*D*_‐value, while with [*P*]_*total*_ >> *K*_*D*_ (i.e., “titration” regime) reliable *K*_*D*_‐values are not reachable. In addition to the hyperbolic model other approaches like the quadratic model can be advantageous. For the case of several ligands binding to one protein, there are further models, for example, described by the Hill equation.

The special feature of fluorescence‐based techniques is that, in principle, signals from both binding partners (P and L) and from the bound complex (P · L) can be measured at the same time. In addition to studies with classical spectrometers and ensemble samples such as fluorescence anisotropy (FA)^[9a]^ or Förster resonance energy transfer (FRET)^[^ ^10^ ^]^ also more advanced techniques on fluorescence microscopes with much lower sample concentrations of nM down to single molecules using fluorescence correlation spectroscopy (FCS),^[^ ^11^ ^]^ single‐molecule FRET (smFRET),^[^ ^12^ ^]^ and two‐color coincidence detection (TCCD) or co‐localization^[^ ^13^ ^]^ are feasible. The latter high‐sensitive techniques in particular have seen significant methodological developments in the recent past, which have opened up interesting and highly biologically relevant areas of application. In addition to their high sensitivity and selectivity (typically the binding partners, or part of them, are selectively labeled with fluorescent dyes), the strength of many fluorescence‐based techniques is that they can be used in complex sample environments. This means that they can be used for quite different in situ applications, for example, in crowded solutions or in bio‐condensates, or in cells. In addition, several fluorescence‐based techniques enable their use with further technical improvements, namely microfluidic approaches.^[^ ^14^ ^]^

In this review, we first introduce some basic principles of fluorescence and explain how fluorescence signals are measured and evaluated for the analysis of binding states. The article also focuses on the potential for binding studies using high‐resolution fluorescence microscopy and spectroscopy. In particular, confocal fluorescence microscopy plays a central role in the analysis of molecules in solution down to single‐molecule sensitivity. Although confocal fluorescence detection and its numerous applications have been discussed in several excellent review articles^[^ ^15^ ^]^ an update of this rapidly developing field with a special focus on high‐affinity binding studies is timely. The inherently low molecule concentration used in high‐resolution fluorescence detection ideally enables the study of high‐affinity binding which plays an important role in numerous cellular processes. These include DNA hybridization,^[^ ^16^ ^]^ protein‐DNA binding,^[^ ^8^ ^]^ protein‐inhibitor binding, and toxin‐antitoxin binding.^[^ ^17^ ^]^ In the latter case, the involved proteins are often intrinsically disordered proteins (IDPs), some of which build up extremely strong binding interactions despite their structural disorder.[^2^, ^12^]

## 2 Fluorescence Principles and Sample Properties

**Figure 1:**
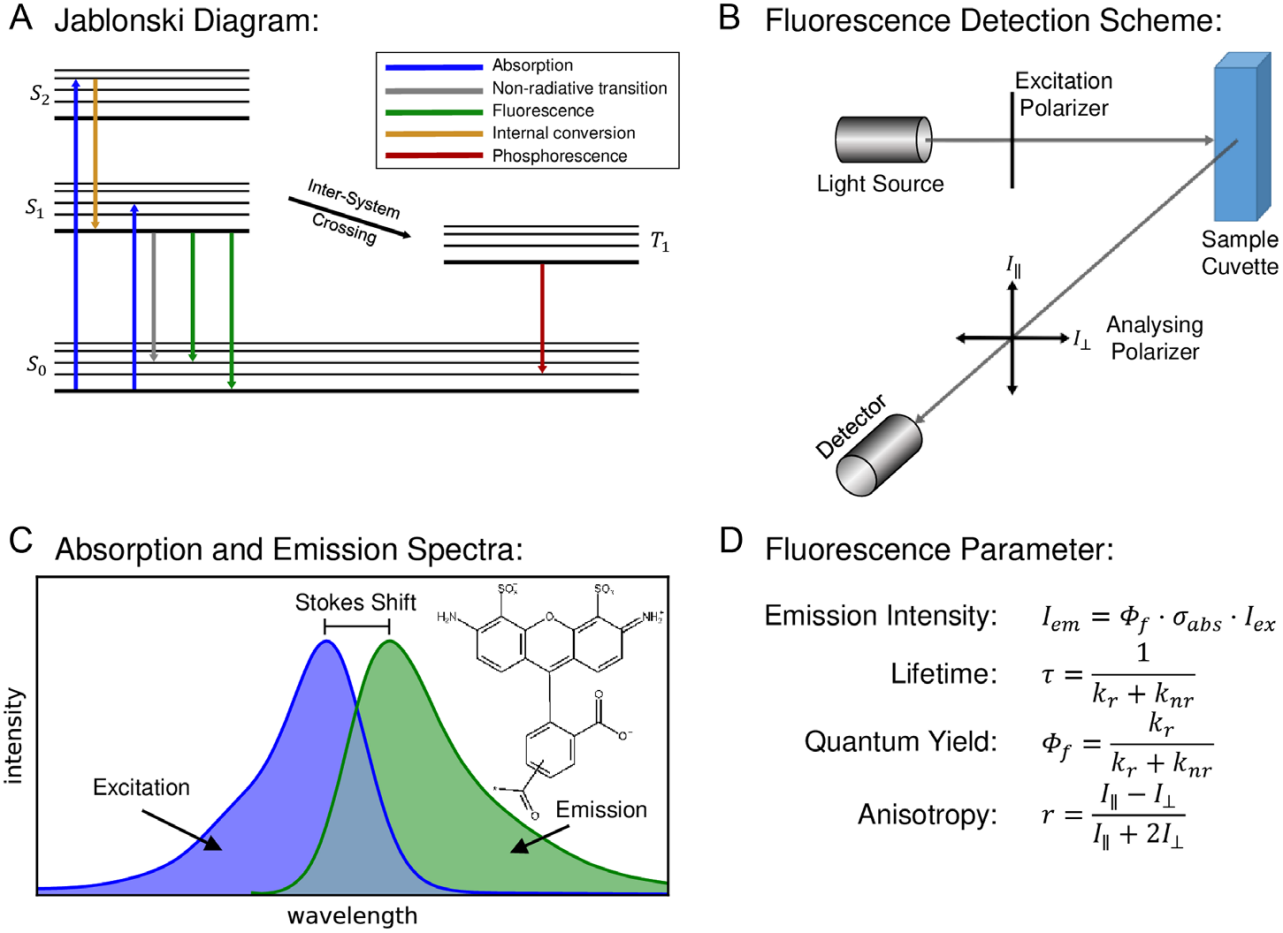
Scheme illustrating fluorescence principles and introducing corresponding fluorescence parameters. A) Jablonski diagram illustrating electronic states (bold lines) and vibrational states (fine lines) as well as possible transitions. B) Fluorescence detection scheme for the measurement of bulk samples in a spectrometer. Typically, the fluorescence emission is measured under 90° angle with respect to the excitation beam direction. For FA measurements the excitation light is linearly polarized and the emission light is measured with an analyzing polarizer allowing light to pass which is either parallel (I_||_) or perpendicular polarized (I_┴_) with respect to the excitation beam. C) Absorption and emission spectrum of a typical fluorophore (inset). D) Definitions of relevant fluorescence parameters.

Fluorescence spectroscopy has long been utilized as a tool to study biological systems, in particular protein–protein and protein–ligand interactions.^[^ ^10^, ^18^ ^]^ Fluorescence occurs when a photon of the incoming radiation is absorbed by a molecule exciting it to a higher electronic singlet state (e.g., S_1_, S_2_,…) followed by emission of light as the molecule returns back to the lowest electronic singlet state S_0_. The emitted light typically has a longer wavelength, and therefore a lower photon energy than the absorbed radiation (**Figure** **1**A,C). In addition to the efficiency of a molecule to absorb radiation of a certain wavelength (determined by the absorption cross‐section σ_abs_), the fluorescence quantum yield ϕ_f_ determines the intensity of fluorescence emission radiation. The process of the transition of the electron back into the electronic ground state shows a molecule‐specific time dependence and is characterized by the so‐called fluorescence lifetime τ, which is usually in the order of nanoseconds^[^ ^19^ ^]^ (Figure 1D). Molecules that absorb radiation in the UV‐vis range and have the ability to fluoresce (fluorophores or dyes) often consist of conjugated π‐electron systems and show a planar molecular structure (see inset Figure 1C). If the fluorophore experiences collisional contacts with surrounding molecules (collisional quenching), the rate of non‐radiative decays (k_nr_) can increase at the expense of the rate of radiative decays (k_r_) and the fluorescence intensity and possibly also the fluorescence lifetime (in the case of so‐called dynamic quenching) can decrease.

The effectiveness of the excitation as well as the fluorescence emission is not only defined by the associated energy levels but also by the relative orientation of the fluorophore with respect to the electric field vector, considered by the transition dipole moment, which is a vector quantity. Information about the rotational mobility of the fluorophores can also be obtained using special experimental setups.^[^ ^19^ ^]^ A scheme how to measure the corresponding fluorescence parameter, namely the fluorescence anisotropy r, is shown in panel Figure 1B.

Since most biomolecules show no fluorescence or (in proteins) only fluorescence via aromatic amino acid side groups (like e.g., tryptophan), a fluorescence labeling of the binding partners under investigation plays an important role in the sample preparation. With a few exceptions, intrinsic tryptophan fluorescence does not play a major role, as it has a relatively low efficiency and can interfere with numerous other biological molecules in the corresponding UV wavelength range (250–350 nm). Therefore, the involved biomolecules are typically modified with fluorophores that yield fluorescence signals at longer wavelengths, with varied fluorescence lifetimes and greater efficiency. In the case of proteins, these are typically labeled side‐specifically on artificially introduced cysteine groups with organic fluorescent compounds (often simply named dye).^[^ ^20^ ^]^ Some of the techniques discussed require marking with two dyes of different wavelengths. There are two approaches to molecule labeling with regard to the protein‐protein interaction to be investigated: (i) labeling only one of the binding partners with both dyes or (ii) labeling both binding partners with one of the dyes each. In addition to approaches with dye attachment, naturally fluorescent proteins with wavelengths in the visible regime are playing an increasingly important role. Numerous variants of the naturally occurring green fluorescence protein (GFP) showing different emission wavelengths from blue to red are nowadays used to monitor cellular components, protein localization, and protein–protein interactions.^[^ ^21^ ^]^ Here in particular the so called genetically encoded FRET biosensors play a central role in monitoring the concentration of metabolites or other small ligands in vitro as well as in vivo.^[^ ^10^, ^22^ ^]^ The sensors are fusion proteins that consist of a central sensing protein flanked by two fluorescent proteins. The optical readout of the sensor is based on FRET between the fluorescent proteins that changes upon ligand binding to the sensing protein.^[^ ^23^ ^]^

## 3 Fluorescence Techniques for Intermolecular Binding and Recent Applications

Based on the use of the respective fluorescence parameters described in the previous section, there are numerous approaches to characterize the corresponding binding properties more precisely. Due to the many different methodological fluorescence‐based approaches, we focus in this article on those methods that detect molecules freely in solutions (**Table** **1**). We are aware that there are also very effective bead‐based and surface methods that are highly sensitive and also allow high throughput.^[^ ^24^ ^]^ All the discussed techniques allow the determination of equilibrium binding constants from the experimental data, whereby the concentration of one binding partner is kept constant and the concentration of the other binding partner is varied within a certain range^[^ ^3^ ^]^ (for details, see also Box 1). Methods (1)–(3) represent well‐established standard techniques that can be performed mainly in classical spectrometers or plate readers. In the case of microscale thermophoresis (MST), there are commercial standard devices that also provide the associated analysis software. The more advanced methods (4)–(6) are performed using fluorescence microscopes and typically require more extensive data treatment and analysis, but also provide greater access to high‐affinity binding and more powerful data. Their methodical details and some recent applications are in the focus of this review and are therefore discussed in more detail than the other methods.

**Table 1:** Overview of fluorescence‐based techniques suitable for bio‐molecular binding studies.

Method	Mode of operation	Affinity Regimea)	References for Applications
(1) Ensemble FRET	Intramolecular FRET; binding‐induced conformational change in one molecule Intermolecular FRET: increased vicinity between both molecules upon binding	10 nM – mM 10 nM – μM	[10, 16, 59]
(2) Fluorescence Anisotropy	Change of the rotational mobility of a smaller molecule upon binding to a larger molecule	10 nM – mM	[9, 60]
(3) Microscale Thermophoresis	Protein/ligand binding to the target molecule induces changes in the thermo‐diffusion	nM –μM	[9, 61]
(4) Fluorescence correlation spectroscopy	FCS (ACF)b): slower translational diffusion of a smaller molecule upon binding to a larger molecule FCCS (CCF)c): high cross‐correlation amplitude if both differently labeled molecules diffuse together (in the bound state)	nM – mM nM – few ten nM	[11, 15, 62]
(5) Single molecule FRET	Intramolecular FRET Intermolecular FRET In solution or surface tethered	pM – μM pM – few 100 nM	[12, 63]
(6) Single molecule two‐color coincidence detection	In solution: Confocal detection applying TCCD/BTCCD analysisd) Surface tethered: TIRFe) imaging with two‐color co‐localization	pM – 10 pM pM – 100 pM	[13, 64]

a) Meaning the regime in which K_D_‐values can be determined.

b) FCS: Fluorescence correlation spectroscopy/ACF: auto‐correlation function.

c) FCCS: Fluorescence cross‐correlation spectroscopy/CCF: cross‐correlation function.

d) TCCD: Two‐color coincidence detection/BTCCD: brightness‐gated two‐color coincidence detection.

e) TIRF: Total internal reflection fluorescence.

### 3.1 Ensemble FRET

**Figure 2:**
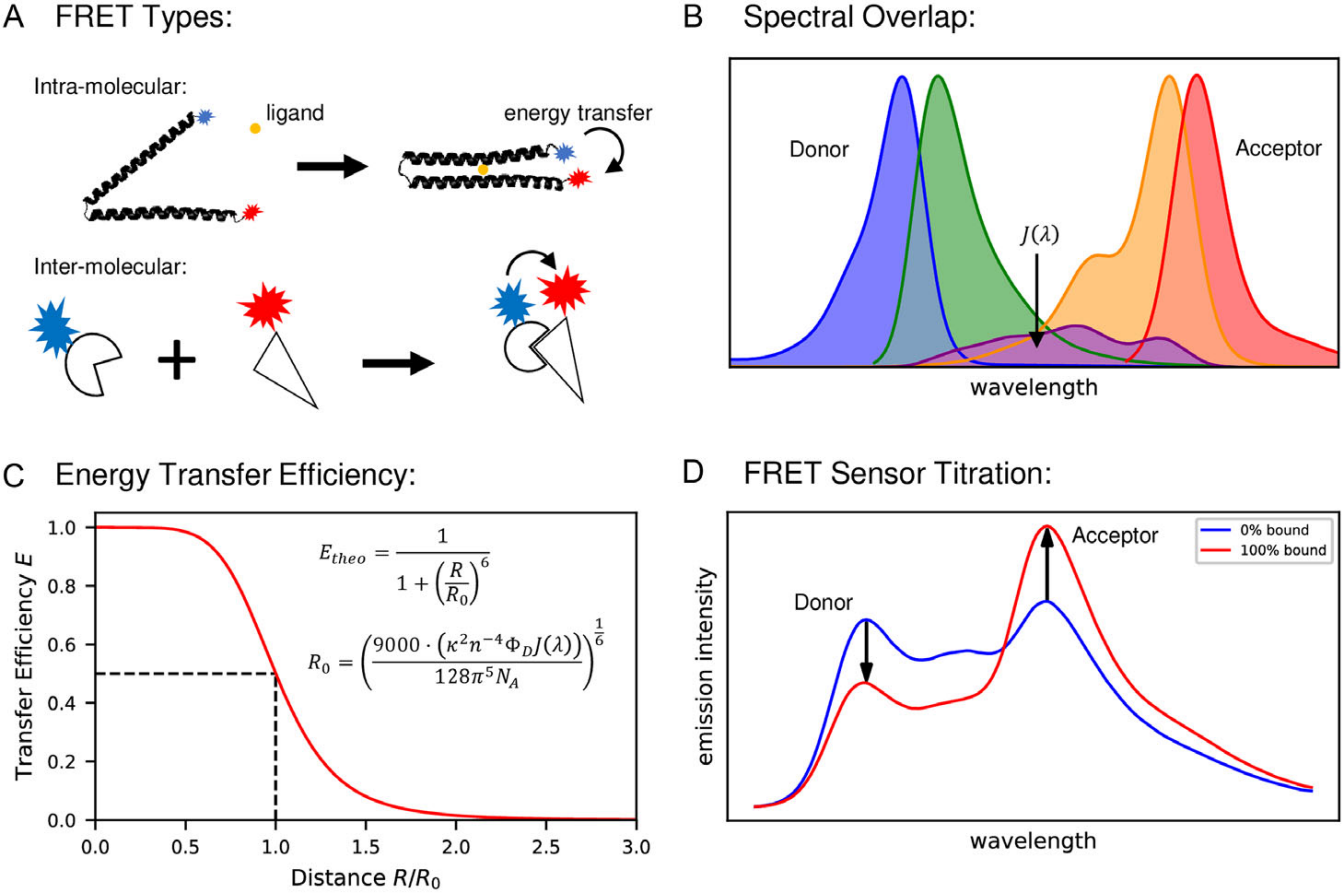
A) Illustration of two different schemes for studying bi‐molecular binding with FRET, (upper panel) a binding‐induced conformational change in one dual‐labeled binding partner (intramolecular FRET) and (lower panel) a binding complex formation with two individually labeled binding partners (intermolecular FRET). The involved dyes are shown in blue color for the donor and in red color for the acceptor. B) Spectra of absorption and emission obtained from donor and acceptor fluorophores as well as the overlap integral J(*λ*) of donor emission and acceptor absorption (violet colored). The latter is needed to calculate the Förster radius R_0_. C) Energy transfer efficiency E plotted as a function of R/R_0_ ratio (with the inter‐dye distance R). D) Exemplary data of an intramolecular FRET sensor for the unbound (blue) and the fully bound (red) state. Here the left peak (donor) decreases at the expense of the right peak (acceptor), which increases upon increasing ligand binding.

After Stryer & Haugland rediscovered FRET as an extremely useful tool for investigating biological topics in their seminal article,^[^ ^25^ ^]^ there have been numerous studies in which FRET has been used to study protein–protein and protein–ligand binding interactions.^[^ ^10^, ^59^ ^]^ This exceptionally effective method to study inter‐molecular binding is possible by using non‐radiative energy transfer between two fluorophores. FRET enables the measurement of distances of a few nanometers and can be used to precisely characterize binding states between the binding partners when applied appropriately (**Figure** **2**A). A prerequisite for this method is the use of two fluorophores in which the emission spectrum of one fluorophore (donor) overlaps with the absorption spectrum of the other fluorophore (acceptor) (Figure 2B).

Since the non‐radiative energy transfer is based on dipole–dipole interaction, the transfer efficiency E shows a strong dependence on distance. This distance R between the two fluorophores can be determined experimentally by exciting the donor fluorophore and relating the acceptor emission I_A_ to the total emission (*I*_*A*_ + *γ* *I*_*D*_) intensity. Here I_D_ is the donor emission intensity and *γ* is a correction factor.

(5) $$E=\frac{I_{A}}{I_{A}+\gamma I_{D}}$$

Another experimental approach for the determination of E is given by

(6) $$E=1-\frac{I_{DA}}{I_{D}}=1-\frac{\tau_{DA}}{\tau_{D}}$$

In this latter approach, the required donor intensities and lifetimes are measured with two different samples, one sample with donor labeling only (*I*_*D*_, *τ*_*D*_) and one with donor and acceptor labeling (*I*_*DA*_,*τ*_*DA*_). According to Förster's theory, E can be assigned to the distance R between the dyes involved.^[^ ^19^, ^26^ ^]^

(7) $$E=\frac{1}{1+{\left(\frac{R}{R_{0}}\right)}^{6}}$$

The distance range in which the resonant energy transfer can be used is determined by the Förster radius *R*_*0*_, which in turn depends on the overlap integral J(λ), the quantum yield of the donor ϕ_D_ and an orientation factor κ,^[^ ^19^ ^]^ for details see Figure 2C. Values of R_0_ for pairs of fluorophores typically used are between two to six nanometers. FRET is mainly used in two different application schemes for analyzing the binding between two molecules. In the first approach, both dyes are bound in one of the two binding partners. If this binding partner undergoes a conformational change due to the binding of the other (non‐labeled) binding partner, this can be detected with the help of FRET. In the other case, both binding partners are labeled with one dye each. Upon binding, that is, spatial approach of the binding partners, a FRET signal can be detected if the attachment positions in both binding partners are suitably selected (see example in Figure 2D).

From a methodical point of view, the choice of a suitable instrumentation is crucial for ensemble measurements as well. Here it is important that the sample of interest is expected to be sufficiently homogeneous to derive useful information from an ensemble average. If not, then single‐molecule FRET may be required. The next question is whether spatial resolution is required. A simple cuvette‐ or microtiter‐plate based measurement is adequate if spatial resolution is not important, whereas FRET imaging is required for spatial resolution, like for “in cell” applications. The latter requires measurements on the microscope. In either case, conventional fluorescence intensity or fluorescence lifetime (to be more precise the donor lifetime τ_D_, see Equation (6)) measurements are possible and can be realized in a cuvette or by fluorescence lifetime imaging microscopy (FLIM). In some cases, fluorescence lifetime measurements can be more reliable than intensity measurements. In particular, they might be useful for obtaining quantitative information when it is difficult to control donor/acceptor concentrations, when a sample has a large or variable scattering background, and when there is interfering fluorescence background. However, a typical trade‐off for lifetime measurements is a more advanced data analysis.^[^ ^27^ ^]^

For practical reasons we will shortly highlight here only two major application approaches. First, in so‐called FRET assays calibration curves are measured, where a decreasing or increasing energy transfer signal is monitored versus the target (binding partner) concentration. In such a spectroscopic measurement either the two binding partners are labeled with the two complementary colors of a FRET pair (i.e., donor and acceptor) at suitable attachment positions so the energy transfer can happen upon binding.[^10^, ^16^] Another approach is the use of FRET biosensors, typically equipped with fluorescent proteins (FPs).^[^ ^28^, ^59^ ^]^ In both cases the measuring and analyzing of the corresponding calibration curves (or binding curves) enables the determination of K_D_ values. With respect to binding studies, FRET imaging can be a powerful as well. Here maps of spatial distribution of proximities between donors and acceptors within biosensors are generated based on images obtained from microscopy. Each voxel in a FRET image is then color‐coded for an energy transfer related value, typically FRET efficiency *E*, the donor lifetime or the intensity ratio *I*_*A*_/*I*_*D*_.^[^ ^29^ ^]^ One challenge of FRET imaging is to acquire enough photons per voxel for accurate measurements.^[59c,59d]^ Furthermore, for valid interpretations of the measured images from living cells multiple control experiments are needed and further sample specific precautions must be taken into account.^[^ ^27^ ^]^

In summary, it can be stated that accurate determination of binding parameters from ensemble FRET measurements is achievable in many cases, but is not straight forward. A range of different sample properties, such as incomplete labeling or background auto‐fluorescence in cells but also methodological complications, such as direct acceptor excitation, cross‐talk, or photo‐bleaching can considerably complicate or prevent error‐free data collection and data analysis. Although there have been considerable methodological improvements in this area in recent times,^[^ ^10^, ^27^ ^]^ another way to avoid these complications as far as possible is to use single‐molecule techniques, namely smFRET (see subsection 3.5).

### 3.2 Fluorescence Anisotropy (FA)

The FA approach, also known as the fluorescence polarization approach, is another important method for analyzing protein–protein or protein–ligand binding. It relies on the differences in rotational mobility of bound versus free ligand. Polarization studies have been used by biochemists for studying protein interactions for more than 50 years as the theory had been thoroughly developed and validated by Francis Perrin and Gregorio Weber. The advent of plate readers significantly increased the use of this method because it introduced a broader community to the rapid screening of a large number of protein–ligand combinations. The method is based on the principle that a fluorophore excited with linearly polarized light emits light that remains partially polarized (see Figure 1B). For excited molecules in solution the degree of remaining polarization is typically characterized by fluorescence anisotropy values between r ≈0.4 (maximal preserved polarization which is typically also referred to as r_0_) and *r* = 0 (100% loss of polarization). Fluorescence anisotropy r is sensitive to factors that affect the rate of the rotational diffusion of the fluorophore and thus depends on the temperature T, the viscosity η of the solution and the apparent size (determined by the volume V of the molecule) of the fluorescent molecule (Perrin equation).

(8) $$r=\frac{r_{0}}{1+\tau /\theta }$$

Here the rotational correlation time is given by *θ* = ηV/RT.^[^ ^19^ ^]^ The apparent size of a protein‐ligand marked with a fluorophore increases when it binds to another binding partner which effectively changes the value of anisotropy. For the most effective assay possible, a fluorescent dye is therefore typically attached to the smaller binding partner. In addition, it is necessary to have a fluorophore with a fluorescence lifetime τ similar to the rotational correlation time *θ* of the molecule of interest. Otherwise, it is not possible to accurately record the difference in anisotropy between a free protein/ligand and one bound to the other binding partner.^[^ ^30^, ^60^ ^]^ Since the anisotropy is an “intensive property” (not depending on the fluorophore concentration) the advantages of FA assays are their simple “mix‐and‐measure” format and the high‐throughput screening capacity when carried out in multi‐well plates.^[^ ^30^ ^]^ However, like most other fluorescence‐based assays, it may also suffer from optical interferences like auto‐fluorescence, quenching, or light scattering.

As shown in recent applications, FA assays can also be used for high‐affinity binding including kinetic parameters (in terms of association and dissociation rates) from time‐resolved measurements. The use of bright and photo‐stable fluorophores (with molar absorption coefficients larger than 100 000 cm^−1^ M^−1^ and quantum yields above 50%), and with fluorescence lifetimes between 3 and 5 ns enables, for example, to study the binding of low‐molecular‐weight ligands to their membrane‐embedded target receptors with K_D_‐values down a few nM.^[60b]^ Another recent achievement of FA‐based applications is making use of fluorescence anisotropy imaging in a dedicated chip reader as part of a microfluidic platform.^[60c]^ More details about microfluidic applications will be discussed in subsection 3.7.

### 3.3 Microscale Thermophoresis (MST)

Microscale thermophoresis (MST) is a powerful method for measuring binding affinities, especially of protein–ligand interactions in free solution, which is widely used in science and industry. The technique is based on binding‐induced changes in the diffusion behavior of molecules. In contrast to FCS, where changes in the size of particles during Brownian diffusion are monitored (see subsection 3.4), MST analyses the thermophoresis generated by temperature gradients in the sample. The advantage of this approach is that not only changes in the size of the molecule or of the molecule/ligand‐complex can be detected but also changes in the charge, the hydration shell, or the molecule conformation. This greatly expands the applicability of the technique for measuring binding interactions.^[9b]^ Since thermophoresis describes a directed movement of molecules in a temperature gradient, a temperature difference in space leads to a depletion of the solvated biomolecules in the region of elevated temperature. This thermophoretic depletion depends on the interface between molecule and solvent. Experimentally thermophoresis is detected in small glass capillaries, which contain a solution where one binding partner needs to be fluorescently labeled. Furthermore, an infrared (IR) laser is focused into the capillary to produce a microscopic temperature gradient spanning a few degrees in a small volume (diameter of few l0 μm). While inducing the temperature gradient, the fluorescent molecules in solution are excited and the thermophoresis dependent depletion (or accumulation) of fluorescent molecules within the temperature gradient is measured. For deriving binding constants, multiple capillaries with constant concentrations of fluorescent molecules and varying concentrations of the ligand are measured consecutively. The related changes in thermophoresis of the fluorescent molecules due to binding to ligand can then be used to determine K_D_‐values.^[61a]^

In the last 10 years, MST has developed into a frequently used standard technology and is applied in numerous areas of the life sciences.^[61a]^ Recently, the technique has been extended for kinetic measurements, enabling the determination of relaxation rates of 0.01–0.5 s^−1^. Stein and co‐workers have characterized association and dissociation rates of hybridizing DNA strands with 10–16 base pairs in solution.^[61b]^ Thus, this technique enables complementary measurements to those obtained with smFRET,^[63c]^ see subsection 3.5.

### 3.4 Fluorescence Correlation Spectroscopy

**Figure 3:**
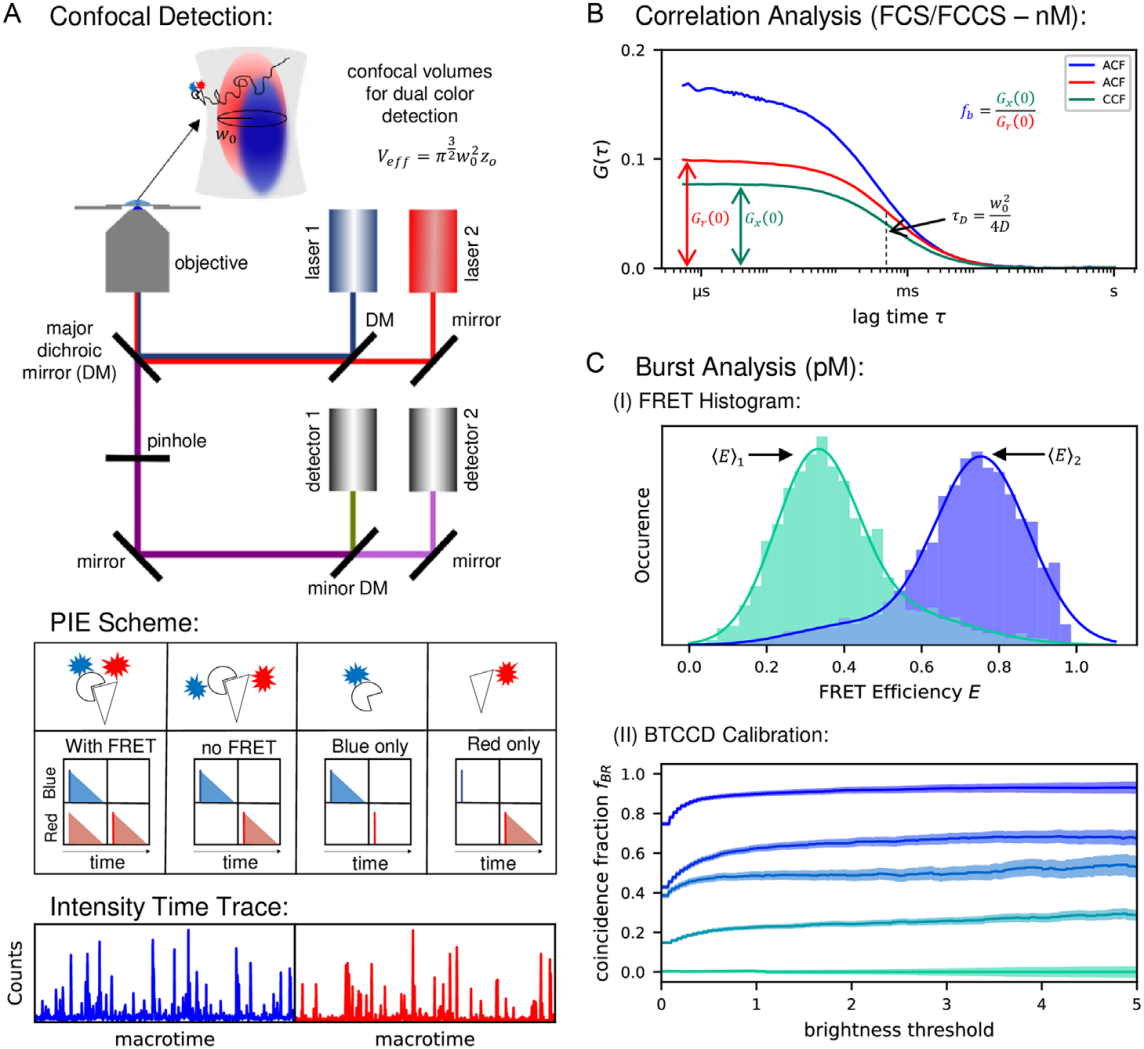
Scheme illustrating confocal fluorescence detection of diffusing particles and data analysis. A) Upper panel: Illustration of a confocal fluorescence setup with pulsed excitation (laser 1,2) and time‐resolved simultaneous dual‐color detection (avalanche photo diode detectors 1,2). By using a pinhole and a high NA objective a confocal detection volume is created which typically differs in size for different excitation wavelengths (e.g., blue and red excitation light). middle panel: Here, blue and red excitations are carried out alternately (with a time shift of approx. 25 ns) and at a repetition rate of 20 MHz, so‐called pulsed interleaved excitation (PIE). Each repetitive interval is characterized by two detection channels (upper, lower subpanel), an excitation pulse (dark blue & dark red bars) and a potential fluorescence emission (light blue & light red triangles). Furthermore, each interval is subdivided into two time intervals: first one with only blue excitation, second one with only red excitation. As shown in the scheme, different scenarios of dye attachments for particles passing through the detection volumes can be distinguished by using this approach. lower panel: Individual (or some) particles which enter or leave the detection volume at given times generate a more or less strongly fluctuating intensity signal as a function of time. Examples of such intensity time‐traces are shown for the two detection channels (blue and red). A similar time‐trace is also generated for the PIE channel (not shown here). B) Using data of intensity time‐traces as measured with molecules in the nM concentration regime, a correlation analysis has been performed. The calculated auto‐correlation functions from blue and red labeled particles and the corresponding cross‐correlation function (green curve) (according to Equation 9 and 12, respectively) are displayed together with relevant parameters (e.g., *τ*_D_ and f_b_) as obtained from fits (Equation 10). C) Data measured with molecules in the pM concentration regime are employed for a burst analysis, which gives access to single‐molecule information. Here, the fluorescence intensity of individual bursts (often visible as separated intensity peaks in the time‐trace) can be employed for two different approaches (smFRET and BTCCD). Upper panel: The obtained burst intensities (i.e., number of photons per burst) are related to the donor and the acceptor channel (I_D_ and I_A_) which are then used to calculate FRET efficiencies E (Equation 5). For hundreds to thousands of bursts the individual E‐values are displayed in a FRET histogram. Here the ligand bound (blue) und unbound (green) subpopulations of a FRET sensor are characterized by their respective <E> values and their statistical weights. Lower panel: In a TCCD analysis the number of bursts measured in both detection channels are used to calculate binding fractions, here for example f_BR_ (see Equation 14). In order to reduce the impact of detection volume mismatch a further brightness threshold is introduced which allows to identify a more reliable f_BR_‐value. This value is reached, when the coincidence fraction as a function of a threshold value shows no further increase with increasing threshold values. In the respective graph data from five different calibration samples are shown, which were preset for defined binding fractions (0%, 25%, 50%, 75%, 100%, for details see^[^ ^41^ ^]^).

Already more than 50 years ago, Webb and co‐workers established a rigorous formalism of fluorescence correlation spectroscopy with its various modes of possible applications. However, only in the 1990s the method became applicable with the advent of laser‐based confocal detection setups which increased the signal‐to‐noise ratio dramatically^[^ ^11^, ^19^ ^]^ (see **Figure** **3**A).

There are some crucial properties of confocal detection and analyzing data for a small number of fluorescent molecules in the detection volume. This applies not only for correlation spectroscopy but also for dual‐color single‐molecule detection, which is used for smFRET and TCCD (for details see subsections 3.5 and 3.6): 1) The required sensitivity for detecting a small number of diffusing particles (even down to single molecules) in solution is made possible by an extremely small fluorescence detection volume (diffraction‐limited), which is generated by using a confocal microscope (Figure 3A); 2) For the discussed fluorescence techniques, the time‐resolved detection, and for two‐color approaches in addition the simultaneous detection of two different emission colors, is essential; 3) Pulsed interleaved excitation, named PIE (with a time delay for a second excitation wavelength), is advantageous for all techniques, which make use of two‐color detection (i.e., FCCS, FRET, BTTCD) to minimize possible measurement errors (Figure 3A).

The simplest approach to use FCS for the determination of binding fractions is to label one binding partner with a dye and detect a change in translational diffusion of that partner caused by binding to the other partner. In practice, this will only be possible if the unlabeled binding partner is larger than the labeled one (at least by one order of magnitude for diffusion in 3D), and thus the labeled partner diffuses significantly slower when bound to the unlabeled partner. Here the diffusion properties are obtained from the measured auto‐correlation function (ACF), given by

(9) $$G\;\;(\tau )=\frac{\left\langle \delta F(t)\delta F\;(t+\tau )\right\rangle }{{\left\langle F\;(t)\right\rangle }^{2}}$$

where δF(t) is the fluctuation of the time dependent fluorescence intensity F(t) signal and δF(t+τ) the fluctuation at a time shifted by a lag time τ (see Figure 3B). For measuring freely diffusing molecules through a Gaussian‐shaped detection volume one can fit the experimental auto‐correlation function with

(10) $$G(\tau )=\frac{1}{N}{\left[1+\frac{\tau }{\tau_{D}}\right]}^{-1}\cdot {\left[1+\frac{\tau }{\kappa^{2}\tau_{D}}\right]}^{-1/2}$$

where N is the average number of molecules in the detection volume, τ_D_ is the average diffusion time through the detection volume and κ^2^ = (z_0_/ω_0_)^2^ describes the spatial dimension of the detection volume (Figure 3A). Typically, the correlation analysis works reasonable well with N ≈ a few up to a few ten, which is related to a concentration range 0.1 – a few 10 nM. On the basis of the obtained diffusion time, the diffusion coefficient D can be determined with τ_D_ = ω_0_^2^/4D, which in turn, with the help of the Stokes–Einstein equation

(11) $$R_{h}=\frac{k_{B}\cdot T}{6\pi \cdot \eta \cdot D}$$

gives access to the size of the diffusing complex via the hydrodynamic radius (R_h_). Several application using this approach are reported in the literature.[^11^, ^62^]

However, the more common and applicable approach to more cases is given by two‐color fluorescence cross‐correlation (FCCS). Here, both binding partners are labeled with dyes of different colors (e.g., blue and red), and a cross‐correlation function (CCF) between both color channels is calculated by

(12) $$G_{x}\;(\tau )=\frac{\left\langle \delta F_{b}\;(t)\delta F_{r}\;(t+\tau )\right\rangle }{\left\langle F_{b}\;(t)\right\rangle \left\langle F_{r}\;(t)\right\rangle }$$

where *δ*F_b_(t) and δF_r_(t+τ) are the fluctuations of the fluorescence intensities measured in the blue and red detection channel, respectively. It is important to note that the CCF at τ = 0 gives the cross‐correlation amplitude G_*x*_(0), which can be used to calculate the binding fractions f_r_ which is a measure of red labeled molecules that have bound a blue labeled molecule. Analogously, f_b_ can also be calculated, see for example, refs. [15, 31] and Figure 3B.

(13) $$f_{r}=\frac{N_{rb}}{N_{r}}=\frac{\frac{G_{x}\;(0)}{G_{b}\;(0)G_{r}\;(0)}}{\frac{1}{G_{r}\;(0)}}=\frac{G_{x}(0)}{G_{b}\;(0)}\;\;and\;similarly\;for\;f_{b}\;=\frac{G_{x}\;(0)}{G_{r}\;(0)}$$

**Figure 4:**
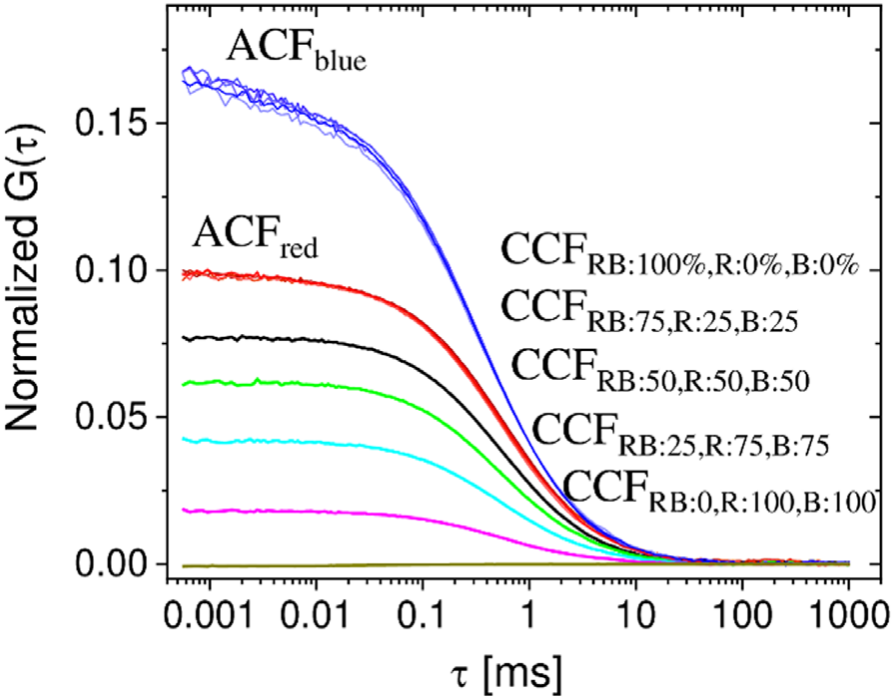
Two‐color FCCS analysis with a set of calibration samples of the same type as those for which data are shown in Figure 2C. Here three different double‐stranded DNA molecules were labeled with a red and a blue dye to varying degrees. RB: dual labeled with red and blue dye, R: single labeled only with a red dye, B: single labeled only with a blue dye. The three different dsDNA molecules were mixed in a way that they all carry the same total concentration of red and blue dyes, but the individual dyes were distributed differently among the double and the single‐labeled dsDNA. The relative percentages are given for all five different mixtures in the legend within the figure. The corresponding CCFs are shown from 100 % dual labeled to 0% dual labeled, from top (black) to bottom (dark green). In addition, the corresponding auto‐correlation functions are shown for the red and the blue detection channel. Although the molecule concentration of red and blue dyes is within the limit of error the same, a lower ACF amplitude at *τ* = 0 is observed for the red channel, because the confocal detection volume is larger than the one in the blue channel. This leads to an apparent higher number of molecules inside the detection volume (G(0) ≈ 1/N). The related (uncorrected, see text) binding fractions can be obtained from Equation 13. However, we clearly see the decrease of the cross‐correlation amplitude with more or less equidistant steps from 100%, 75%, 50%, 25% to 0% dual‐labeled species.

Here G_r_(0) and G_b_(0) represent the auto‐correlation amplitudes at τ = 0 (Equation (9)) for the red and the blue channel, respectively. This efficient and straight‐forward approach can be well illustrated using a suitable set of calibration samples (**Figure** **4**).

However, various artifacts can distort the values of a measured binding fraction and must therefore be corrected if quantitative results are required. The data in Figure 4 show, for example, that the maximum cross‐correlation amplitude for a perfect sample (completely double‐marked, black curve) is not reached, as G_x_(0) ≠ G_r_(0) which we would expect for a perfect sample (with f_b_ = 1). One of the artifacts causing incorrect values for the obtained binding fractions is given by the mismatch of the detection volume in two‐color detection. The detection volumes of the two color channels with different wavelengths are (i) different in size and (ii) typically shifted against each other due to chromatic aberration (Figure 3A). In addition to the detection volume mismatch, incomplete molecule labeling, unwanted Förster resonance energy transfer, photo‐bleaching, or cross‐talk can falsify the measured cross‐correlation amplitude and thereby the obtained K_D_‐values. To account for some sources of error one possible strategy is to make use of PIE‐FCCS. In pulsed interleaved excitation (PIE),^[^ ^32^ ^]^ for example, the excitation color is alternated every 25 ns (see Figure 3A). This means that each molecule that diffuses through the detection volume (with dwell times in the millisecond range, depending on the size of the molecules) is excited 10^4^ to 10^5^ times, resulting in a sufficiently high number of photons in the respective color channels per molecule transit. However, it is important to note here that special calibration measurements are usually necessary to ultimately obtain reliable K_D_ values for the intermolecular binding under investigation.^[^ ^15^, ^33^ ^]^ This applies in particular to measurements in cells (in vivo), which are generally much more demanding and error‐prone than in vitro measurements.^[^ ^34^, ^62^ ^]^

The ability of FCS techniques to study relevant molecular processes in living cells has been convincingly demonstrated in several recently published studies. A first example deals with the assembly and disassembly of cyclin B1 and Cdk1, which form a mitotic kinase complex. A combination of FCS and FCCS measurements revealed that the affinity of the binding partners involved is increasing during the cell cycle, indicating that the assembly of the complex is a regulated step.^[62c]^ In another case, the EphA2 receptor for membrane‐bound ephrin ligands in cancer cells was investigated with time‐resolved live‐cell spectroscopy. EphA2 was characterized with respect to diffusion, oligomerisation, and conformational changes. Here, PIE‐FCCS was used to reduce the likelihood of false positive cross‐correlation, a major problem with fluorescent protein (FP) probes and in rather heterogeneous systems like the plasma membrane. The experiments revealed that EphA2 forms multimers through two different modes of interactions. One mode drives ligand‐induced receptor clustering leading to tumor suppressive signalling, while the other mode supports oncogenic signaling.^[62d]^ Both examples convincingly show that high temporal and spatial resolution is the key to studying the dynamics of protein complexes in living cells.

Depending on properties of the confocal setup (wavelength, pinhole size), FCCS can be conducted at molecule concentration (of labeled species) in the subnano‐molar to the micro‐molar range, which would give access to K_D_‐values for binding studies from nM to maximal 100 nM. In the case of confocal detection, the FCCS technique shows an overlap with ensemble FRET and the FA approach for the weaker binding interactions. For the stronger bindings, it covers K_D_ values 100 times larger than those measured with the TCCD technique and the smFRET approach (with both binding partners dye‐labeled), see subsection 3.5 and 3.6.

### 3.5 Single Molecule FRET

FRET‐based studies with single‐molecule sensitivity were first carried out with biological macromolecules under physiological conditions in the 1990s. Mainly conformations of proteins during the folding transition or functional conformational states were analyzed.^[^ ^15^, ^35^ ^]^ From a methodological point of view, two different approaches were mainly used for smFRET studies:^[35c]^ (i) confocal fluorescence detection of freely diffusing molecules and (ii) total internal reflection fluorescence (TIRF) microscopy with molecules immobilized on the surface.

In intensity‐time traces measured with molecules in the picomolar concentration range, the essential fingerprint in the confocal fluorescence detection of diffusing molecules is the so‐called “photon burst” as the central measured variable for a single molecule (see Figure 3A). The fluorescence intensities of the donor and the corresponding acceptor burst for a single dual‐labeled molecule diffusing through the detection volume are used to calculate the corresponding FRET efficiency E (Equation (5)) of a single molecule. For measurements over longer times (minutes‐hours), hundreds to thousands of single molecule FRET efficiencies *E*_*i*_ can be obtained from such intensity time traces. The obtained efficiency values will then be displayed as a histogram and represent the distributions of the coexisting subpopulations, as shown in Figure 3A for an example of a bound and an unbound state.^[^ ^36^ ^]^ Each subpopulation in the histogram is typically fitted with a Gaussian, that is characterized by an efficiency mean‐value <E> and a statistical weight of the subpopulation. The latter, as obtained from the normalized area under the curve of the Gaussian fit, is directly related to the binding fraction if the sample represents a binding assay.

For smFRET, most binding studies were performed with a dual‐labeled binding partner (intramolecular FRET) and an unlabeled ligand. In this case, the ligand must induce a measurable change in the structure of the dual labeled binding partner, which serves as a signature for a binding event. It is advantageous here that the unlabeled ligand can be titrated in more or less unlimited concentration ranges without interfering with the smFRET measurement.[^12^, ^63^] It is much more challenging to perform smFRET studies with two individually labeled binding partners, in studies using confocal detection with diffusing molecules (intermolecular FRET). Since the latter usually have to be carried out with molecular concentrations of the order of picomolar, binding studies are only possible with partners that have extremely high binding affinities (for more details see “concentration barrier” problem^[^ ^15^, ^37^ ^]^ and discussion in subsection 4). On the contrary, the approach with both binding partners labeled allows direct monitoring of the actual concentration of both binding partners in the solution. The latter is of particular importance at very low concentration where unspecific binding of involved molecules can cause inconsistencies in titration measurements.^[^ ^38^ ^]^ In the case of smFRET studies where one binding partner is tethered to the surface of a cover‐slide (ideally labeled with the donor), the range of possible binding affinities can be significantly larger, since the other binding partner (labeled with the acceptor) is added to the buffer can be in a concentration regime up to 100 nM. Here the single molecules (binding pair) are usually imaged by a TIRF microscope. This approach partially circumvents the above‐mentioned “concentration barrier” problem and is therefore probably most commonly used for smFRET binding studies.[^12^, ^63^]

**Figure 5:**
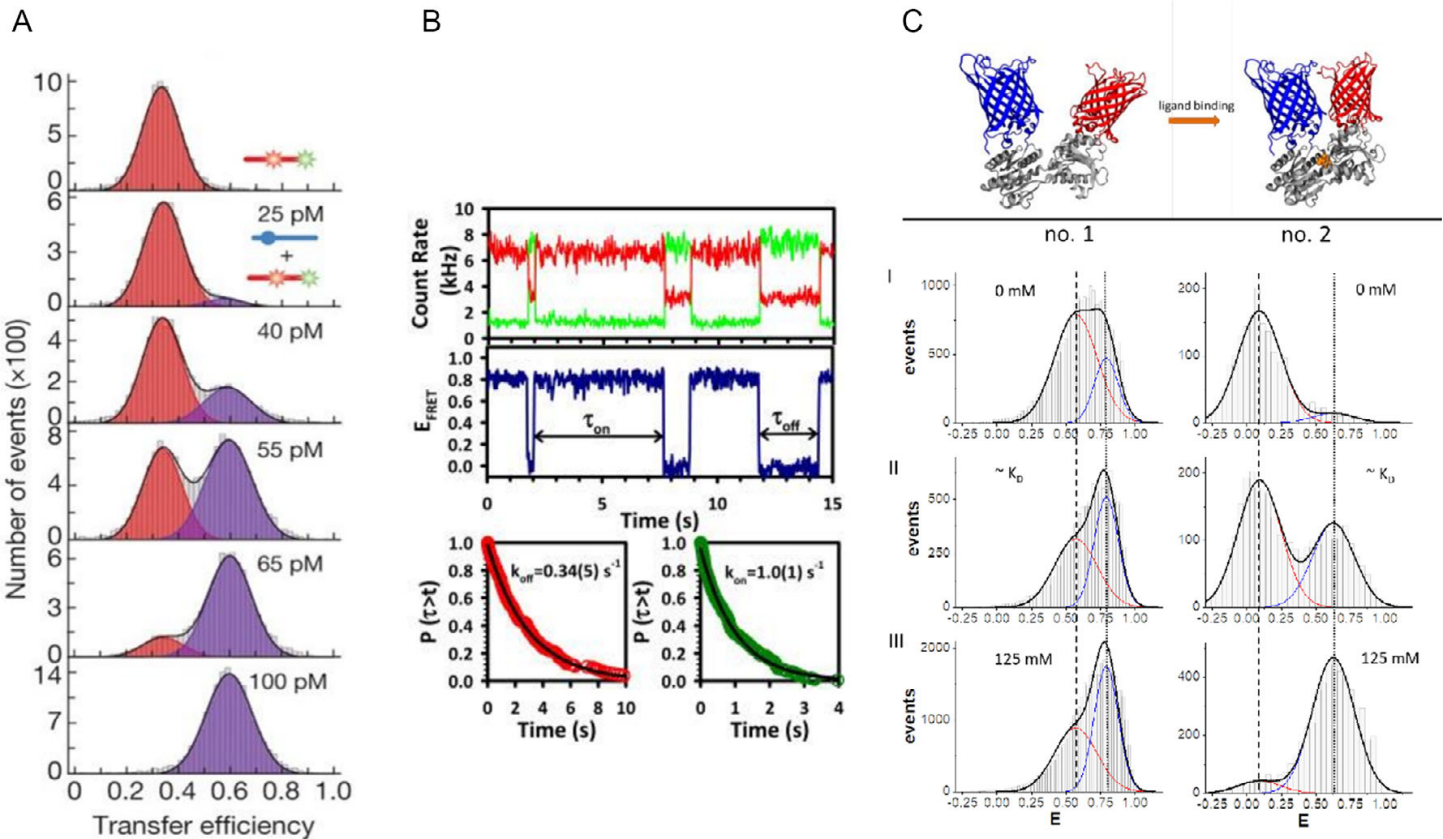
Examples of binding studies employing smFRET. A) FRET efficiency histograms of dual‐labeled ProTa molecules and unlabeled H1 with varying concentrations (between 0 and 100 pM). Here the red colored peak represents the unbound population and the blue colored one the bound population. Based on the data shown, a resulting K_D_‐value of ≈50 pM was obtained for the given ionic strength. Adapted with permission from^[12b]^ Copyright 2018, Springer Nature. B) Binding and unbinding events are observed as anti‐correlated intensity fluctuations between the donor (green) and the acceptor (red) channels (upper panel). In the unbound state the acceptor intensity does not drop to zero, due to the presence of multiple freely diffusing acceptor labeled single strands. From the intensity traces FRET values can be calculated, which exhibit a high and a low FRET state, that is, the bound and unbound state, respectively (middle panel). Crossings between high and low FRET state define a time (dwell time) in which the construct stays in the corresponding state. The measured dwell times are used for fittings with a probability distribution function to obtain association rates (k_a_ or k_on_) and dissociation rates (k_d_ or k_off_), see lower panel. Adapted with permission from^[63c]^ Copyright 2013, Elsevier. C) The working principle of a FRET‐based biosensor is illustrated in the upper panel. Upon ligand binding the relative distance between the involved FPs decreases and the related FRET efficiency increases. Two different sensor constructs (no. 1 and no. 2) are compared with respect to their performance. The corresponding FRET histograms display a bound population (blue colored peak fit) and an unbound population (red colored) at different ligand concentrations, from to top to bottom: (I) no ligand, (II) concentration around the K_D_ value of ≈1 mM, and (III) fully saturated). Adapted with permission from^[^ ^36^ ^]^ Copyright 2018, ACS.

In a first example (**Figure** **5**A), the application of smFRET elucidates the character of an ultrahigh‐affinity binding between two intrinsically disordered proteins (IDPs). In contrast to the existing paradigm that specificity and high binding affinity requires a certain precise complementarity of the structure of the binding interface, the authors report an interaction between two unstructured proteins, namely ProTα and H1, with a K_D_ –value in the picomolar range.^[12b]^ This study revealed that mainly large opposite net charges of the two proteins drive the strong interaction, while neither defined binding sites nor specific individual amino acid residues play a relevant role.

In another application, oligonucleotide duplex hybridization was investigated on a confocal microscope (Figure 5B). Both interaction partners, here single stranded DNA (ssDNA), were labeled individually with dyes of different color, where one single strand was tethered to the surface of a cover slide, while the other single strand was added to the solution. Only upon binding, i.e., hybridization to a double stranded DNA, (dsDNA), donor and acceptor dyes come into a distance below the Förster radius and FRET takes place. This process of association and dissociation of dsDNA can be monitored on single‐molecule level at thermodynamic equilibrium by measuring intensity time traces.

These intensity time traces can be used to determine association rates k_a_ and dissociation rates k_d_ and thus K_D_ values (see Box 1). In their study Dupuis and co‐workers investigated the hybridization kinetics for different numbers of complementary base‐pairs (6 – 9 base pairs), at different NaCl concentrations, and at different temperatures.^[63c]^ Overall, such a comprehensive study gives valuable insights about how this biological most important interaction depends on physiological parameters.

Finally, genetically encoded FRET biosensors were studied with smFRET in order to elucidate the sensor performance of newly developed sensor constructs. Typically, these sensors are equipped with two fluorescence proteins (FPs) constituting a FRET pair (e.g., CFP and YFP) which enclose a ligand binding element (sensing domain), see subsection 2. As shown in Figure 5C smFRET data which is displayed in the corresponding FRET histograms give access to ligand bound and unbound populations which show clear differences for different sensor constructs under investigation (here no. 1 no. 2). The successful design of a sensor construct depends mainly on the fact how efficient a conformational change in the sensing domain is transferred to a distance change between both FPs. The comparison clearly shows that construct no. 2 works much better since the bound and unbound populations are better separated (in terms of mean peak position shift) and the individual population are more fully populated or depopulated under their respective ligand concentration (recognizable by the statistical weight of the corresponding population, i.e., the area under the related curve).^[^ ^36^ ^]^ Ultimately, the performance of the sensor depends on the most appropriate insertion position of the FPs in the amino acid sequence of the sensor domain and on the linker sequence introduced between the FPs and the sensor domain.^[10b]^ Although the final application with suitable sensors is typically done using ensemble techniques (see subsection 3.1), single molecule techniques in addition to other methodological approaches can be very helpful to support the design process.^[^ ^39^ ^]^

### 3.6 Two‐color Coincidence Detection

The idea of single molecule two‐color coincidence detection (simply abbreviated here by TCCD) was already present at the beginning of the first smFRET studies, since TCCD measurements are technically very similar to those of smFRET in solutions. On the other hand, similar questions were addressed with a correlation approach between signals of two colors, a technique that was already used in the 1990, named two‐color fluorescence cross‐correlation spectroscopy^[11b]^ (see subsection 3.4). However, in TCCD analysis, individual molecules are identified via bursts in the respective color channels. If two bursts coincide in time, it can be assumed (at low molecule concentrations) that two molecules, each labeled with dyes of different colors, diffuse together through the detection volume and are therefore bound to each other (see Figure 3A). The ratio of the number of burst for measured particles which emit photons of color 1 (e.g., blue) simultaneously with photons of color 2 (e.g., red), given by N_BR_, with respect to all bursts of color 1, given by N_B_, represents the binding fraction.

(14) $$f_{BR}=\frac{N_{BR}}{N_{B}}\;and\;f_{RB}=\frac{N_{RB}}{N_{R}}$$

**Figure 6:**
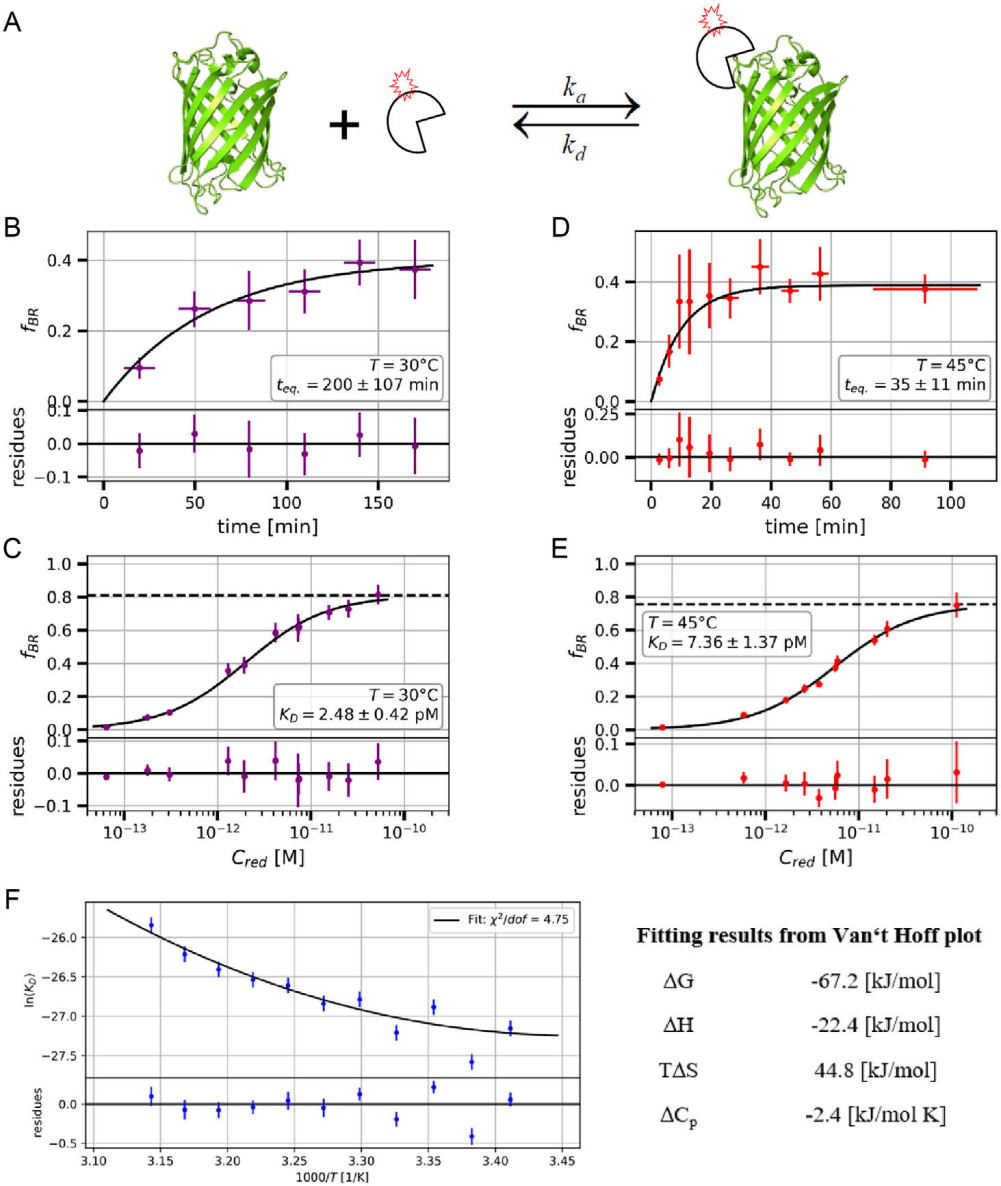
Applications of BTCCD for an antigen‐antibody binding study. A) The antigen is represented by an intrinsically fluorescent protein, an EGFP, which is suitable for single‐molecule studies. The corresponding antibody (nanobody) is labeled with a red fluorescent dye. B,D): Depending on the environmental temperature, the equilibrium time for complete binding (at given molecule concentrations) is much shorter for 45 °C as compared to 30 °C. C,E): Single molecule‐based titration measurements were used for fitting a binding curve (hyperbolic fit model, see Box 1) which enables the determination of the corresponding K_D_‐values. As expected the binding interaction is slightly weakened at higher temperatures. F) Van't Hoff plot with K_D_ values from obtained f_BR_ values as measured at temperatures between 20° and 45 °C. The fit (black line) of data points in the plot results in the given thermodynamic parameters at a reference temperature of 25 °C. Adapted with permission from Schedler et al.^[13b]^ Copyright 2023, MDPI.

Equivalently, the complementary binding fraction f_RB_ can also be determined from the same measured data. The obtained binding fractions can directly be used to determine a K_D_ value by performing a titration assay, see Box 1. In comparison to the intermolecular smFRET approach, the TCCD method does not require the dyes to be attached at positions suitable for FRET. In this respect smFRET is often more challenging, because detailed knowledge about the molecular structures of both binding partners is required. Furthermore, dyes attached for the FRET approach are typically need to be quite closed to each other (≈5 nm) which may cause problems if the dyes directly exhibit attractive or repellent interactions within the binding interface. From the technical point of view, application of the PIE scheme allows the suppression of some sources of error in data acquisition (e.g., unwanted excitation of dyes of the other color, unwanted FRET). Importantly, the TCCD approach works most efficiently at molecule concentrations in the range of a few picomolar when using diffraction‐limited detection volumes as in a confocal microscope. At higher molecular concentrations, chance coincidences for the simultaneous occurrence of bursts of both colors, simply caused by individual molecule diffusion, are no longer negligible. Although specific corrections can partly circumvent the problem,^[^ ^13^, ^40^ ^]^ in general, however, at concentrations well above several 10 pM the TCCD method can no longer be used to determine reliable K_D_ values.^[^ ^38^ ^]^ Another important shortcoming of the TCCD approach is the mismatch of the detection volumes in two‐color detection. This leads to a systematic underestimation of the measured binding fraction, a phenomenon that has also been observed with dual‐color FCCS[^15^, ^33^] (see subsection 3.4). In the case of the TCCD approach at the single molecule level, the introduction of an additional brightness threshold can circumvent the problem mentioned. If only bursts with a “high” brightness are selected, these are typically caused by molecules that diffuse through the centre of the respective detection volume. Thus, there is a very high probability that the molecules considered in the data evaluation have diffused through the detection volumes of both wavelengths (Figure 3A). This approach, brightness‐gated TCCD (BTCCD), enables the determination of reliable binding fractions, as shown with the help of suitable calibration samples^[^ ^41^ ^]^ (Figure 3C). To demonstrate the reliability of the BTCCD method for the determination of high affinity binding, the bi‐molecular binding between an enhanced green fluorescence protein EGFP (antigen) and the related fluorescently labeled nanobody (antibody) was measured (**Figure** **6**A). In contrast to conventional antibodies, nanobodies consist of a single polypeptide chain (≈14 kDa), but bind to their targets with similar affinities as full‐length antibodies.^[^ ^42^ ^]^

As shown in Figure 6 the binding of this antigen‐antibody pair has a binding affinity with a K_D_‐value in the low picomolar regime. The measured data not only show an excellent statistical reliability for measurements with such low molecule concentrations, but also a pronounced temperature dependence of the binding affinity. More methodical details were described elsewhere.^[13b]^ Measurements as a function of temperature (on the confocal microscope in practice in the range between 10°–45 °C) also allow the extraction of relevant thermodynamic parameters (Δ*H*, Δ*S*, Δ*C*_p_) which enable a detailed characterization of the binding interaction (Figure 6F).^[2a−c]^

### 3.7 Applications with Microfluidic Devices

Today, microfluidic devices can routinely handle liquids in the pico‐ to nanoliter range. The dimensions of these micro‐scale devices enable the manipulation, mimic and measurement of biological processes with a resolution that was not possible on a macro scale with classic bulk samples.^[^ ^43^ ^]^ Microfluidic techniques are therefore well suited for the investigation of protein‐protein and protein‐ligand interactions under conditions that approximate the native, i.e., cellular, state.^[14c]^ Since the field of application in the life sciences is almost infinite, we will limit ourselves here to a brief presentation of some conceptual approaches that are directly related to the binding properties of biomolecules and the preceding techniques.

**Figure 7:**
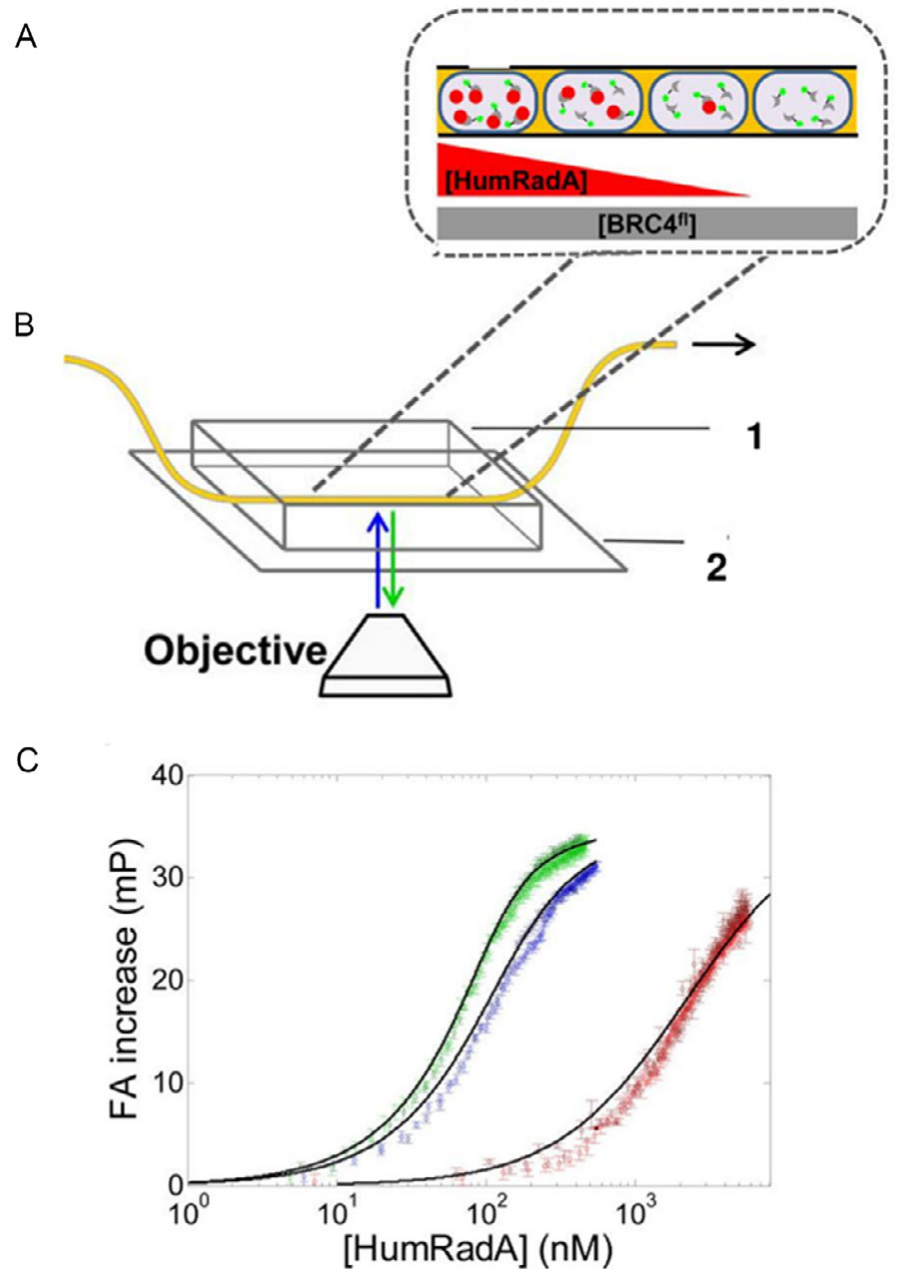
Experimental realization of a quantitative binding assay. A) Scheme of the titration in droplets. The droplets are produced to set up a concentration gradient of the respective ligand (HumRadA), so that the ratio of HumRadA to the other binding partner (BRC4) increases. B) Schematic view of the fluorescence anisotropy detection system developed for affinity determination. Microdroplets are inserted in an inert tubing (yellow). The 10 – 20 nL droplets encapsulate the aforementioned concentration gradient. The microchannel device is made of a silicone polymer (1) and is bonded to a coverslip bottom (2), while the droplets in the microchannel are imaged by a microscope. C) Titration of BRC4 with three different HumRadA variants (colored data points) at constant 100 nM BRC4. K_D_ values were extracted from the fits (black lines) Adapted with permission from Gielen et al.^[^ ^47^ ^]^ Copyright 2017, ACS.

(i) Since fluid behavior on the microscale differs greatly to that of bulk solution, fluid mixing in microfluidic channels can facilitate strategies that are not feasible in the bulk phase. Microfluidic devices enable for example the investigation of low‐affinity binding interactions with confocal single‐molecule spectroscopy. A corresponding study reports that a microfluidic device allows a concentrated sample to be diluted by up to five orders of magnitude within milliseconds, at the physical limit dictated by diffusion.^[^ ^44^ ^]^ Another issue is related to the fact that reliable binding curves need to be measured with titrant concentrations that must vary over 2–4 orders of magnitude. Based on a tree structure of microfluidic channels of different channel diameters, logarithmic concentration gradients can be produced quickly and reliably with very small sample volumes.^[14b]^ Such approaches are ideal for high‐throughput measurements where the binding properties need to be quantified under a wide range of different environmental conditions.

(ii) In addition to mono‐phase liquids in microfluidic channels another approach makes use of an aqueous liquid phase which is inserted into an immiscible carrier fluid (typically an oil). In such a way water‐in‐oil emulsion droplets are formed which are made and handled in specialized microfluidic devices. The droplet compartments with pico‐to nanoliter volumes replace the classical test‐tubes or multiwall‐plates and allow a minimal consumption of reagents and plastic ware.^[^ ^45^ ^]^ Importantly, the encapsulate droplets can mimic artificial cells or isolated reaction vessels and therefore represent an extremely efficient platform to study bio‐molecular binding interactions in a highly controllable and rapid manner.^[14a]^ In recent years, numerous highly pioneering studies have been carried out using the microfluidic droplet approach.^[^ ^46^, ^60^ ^]^ One example is highlighted here where a protein‐protein association is analyzed by fluorescence anisotropy measurements in nanoliter droplets (**Figure** **7**). This study reports the yield of high‐resolution binding curves obtained from tens of microliters reagents, titrations spanning 2 orders of magnitude in concentration, and ≈100 data points measured per minute.^[^ ^47^ ^]^

(iii) A further aspect of microfluidic channels that is more relevant for the confocal fluorescence microscopy method is connected with the possibility of reducing the detection volume through the physical confinement of diffusing molecules through submicrometer‐sized fluidic channels. Here the effective observation volumes created by these channels are ≈100 times smaller the those using only conventional confocal optics and thus enable molecule detection at higher concentration.^[^ ^48^ ^]^ As discussed in subsection 3.5 such an approach can help in breaking the concentration limit of optical single‐molecule detection.^[15g]^ However, channels with dimensions of a few micrometers and less have their own challenges, such as increased surface adhesion and clogging.

## 4 Summary and Outlook

In this review, numerous examples showed the possibilities of fluorescence‐based measurement techniques for investigating the bi‐molecular binding of biological molecules. In these reports, mainly in vitro studies were presented, as these are typically open systems that allow a controlled change in ligand concentration and thus make it possible to measure binding curves. Measurements in (living) cells, on the contrary, often only allow qualitative analyses of binding interactions in a certain affinity range. Nevertheless, such studies can be already extremely informative and can provide deep insights into cellular processes. However, the FRET biosensors discussed in this review can in principle be used to obtain quantitative results from measurements in living cells. If such FRET biosensors are first used to perform calibration curves in open in vitro assays with controlled varied ligand concentrations, a quantitative readout of the ligand concentration present in the cell can be performed with the help of the calibration curve obtained. Unfortunately, the straightforward application of this approach often still suffers from some practical difficulties. First, the cellular milieu typically shows a more or less pronounced crowding effect, which leads to distorted readout values in many FRET sensors that function according to the venus fly‐trap principle.^[^ ^36^, ^49^ ^]^ Secondly, FRET biosensors are limited by their intrinsic detection range, which is usually only two orders of magnitude of the analyte concentration.^[^ ^50^ ^]^ Here so‐called intensiometric single‐FP^[^ ^51^ ^]^ or ratiometric matryoshka^[^ ^50^ ^]^ biosensors with a much larger dynamic range can be advantageous, as they do not use FRET and appear to be less susceptible to crowding effects.

Another important aspect of the application of fluorescence‐based techniques is the extension to binding studies covering the entire range of high‐affinity binding. In particular, studies in which both binding partners are labeled show a gap between a few 10 pM to a few nM with regard to measurable K_D_ values in typical measurements with diffraction limited detection volumes (see Table 1). In order to close this gap in the accessible K_D_ values, the approach of reducing the detection volume offers some methodical solutions.^[15g]^ In addition to the use of two photon excitation and stimulated emission depletion (STED),^[^ ^52^ ^]^ this also includes the use of nanofluidic (see subsection 3.7) and nano‐apertures, such as the so‐called zero mode wave guides (ZMWs).^[^ ^53^ ^]^ However, the latter technique requires one binding partner to be surface‐anchored, but allows for example smFRET studies with concentrations of the ligand up to mM.^[37b]^

Although the dual labeling of interaction partners in binding assays is more time‐consuming and methodically more demanding than other standard techniques, it offers important advantages in the investigation of intermolecular binding. In the case of individual labeling of both binding partners, this approach offers the unique opportunity to (i) monitor the actual concentrations throughout the duration of the experiment and (ii) directly distinguish between free and bound species (see Box 1). This makes it possible to determine very reliable K_D_ values in the range of a few pM.^[13b]^

In assays in which one binding partner is fluorescently dual labeled, not only binding can be detected via intramolecular FRET (binding‐induced conformational change), but such samples are also suitable for providing further information on conformational dynamics. This has been used extensively in the case of IDPs. In numerous cases, high‐affinity binding was found for these unstructured proteins. Due to the lack of a stable spatial structure, tight binding is not only made possible by “short linear motifs” of 3–5 residues, but also through the presence specific dynamic properties of the polypeptide chains involved.^[^ ^54^ ^]^ Since IDPs often do exist in a variety of conformations, a single IDP can interact with many different partners. Studies have shown that IDPs and proteins with intrinsically disordered regions (IDRs) play central roles in protein networks, in particular, acting as hub proteins for molecular communication via protein–protein interactions (PPI).^[^ ^55^ ^]^ Upon binding to target proteins, IDPs become structured to varying degrees, from fully ordered complexes, in which α‐helices are usually formed, to those that exhibit a high degree of disorder in the bound state, the so‐called fuzzy complexes. Obviously, the process of binding can be coupled with a significant loss in conformational entropy of the IDP/IDR. However, if for example the gain in enthalpy coupled with folding is sufficient to pay the entropic penalty of binding, the overall process of binding is feasible.^[2d]^ Studies in this direction are ideally covered by BTCCD measurements, as shown in subsection 3.6 (see Figure 6F). It can therefore be concluded that fluorescence‐based single‐molecule techniques can make an important contribution here and are already helping to elucidate new binding mechanisms in structural biology.^[^ ^2^, ^12^, ^13^, ^56^ ^]^

Probably the greatest potential for overcoming the current limitations in performing most powerful binding studies lies in the rigorous development of the interface between sample preparation and signal detection to determine the binding fraction. Part of the limitation is that for a more complete characterization of the macromolecular binding, binding fractions should be measured at numerous different environmental conditions. In addition to temperature dependence (to determine thermodynamic parameters, see section 3.6), pH values, ionic strength and crowding effects often play an important role. In practice, this represents a very large parameter space for which most measurement methods require extremely long measurement times and time‐consuming sample preparations. Automated platforms can help to overcome this problem, as recently demonstrated in the case of smFRET studies using multi‐well plates.^[63b]^ The use of microfluidic devices should represent an even greater potential for high‐throughput studies (as already discussed in section 3.7). This means that further new functions can be realized on microfluidic chips, especially for confocal microscopy applications. Some latest developments describe, for example, an electrophoretic sorting option for molecules in a bound and unbound state^[^ ^57^ ^]^ or the production of ligand dilutions over 4 orders of magnitude in concentration. Such strongly extended concentration ranges are essential for conclusive determinations of K_D_‐values and can be realized with logarithmic concentration gradient generators.^[14b]^ In addition to the ability to handle a sequence of multiple subsequent steps in sample treatment on the microfluidic chip also the sample detection need to be multiplexed. The confocal detection principle can, for example, be extended to a highly parallel optical system that can analyze up to hundreds of sample points simultaneously.^[^ ^58^ ^]^ Combining many of these options would significantly reduce the effort for sample preparation and the corresponding measuring time and would give access to much more complete data as well as to new and more challenging research topics.

## Conflict of Interest

The authors declare no conflict of interest.
